# Cyclic GMP-Dependent Regulation of Vascular Tone and Blood Pressure Involves Cysteine-Rich LIM-Only Protein 4 (CRP4)

**DOI:** 10.3390/ijms22189925

**Published:** 2021-09-14

**Authors:** Natalie Längst, Julia Adler, Olga Schweigert, Felicia Kleusberg, Melanie Cruz Santos, Amelie Knauer, Matthias Sausbier, Tanja Zeller, Peter Ruth, Robert Lukowski

**Affiliations:** 1 Department of Pharmacology, Toxicology and Clinical Pharmacy, Institute of Pharmacy, University of Tuebingen, 72076 Tuebingen, Germany; natalie.zinn@uni-tuebingen.de (N.L.); julia.straubinger@uni-tuebingen.de (J.A.); feliciakleusberg@gmail.com (F.K.); melanie.cruz-santos@uni-tuebingen.de (M.C.S.); amelie.knauer@gmail.com (A.K.); matthias.sausbier@uni-tuebingen.de (M.S.); 2 Cardiovascular Systems Medicine and Molecular Translation, University Center of Cardiovascular Science, University Heart & Vascular Center Hamburg, University Medical Center Hamburg-Eppendorf, 20251 Hamburg, Germany; o.schweigert@uke.de (O.S.); t.zeller@uke.de (T.Z.); 3 DZHK, German Center for Cardiovascular Research, Partner Site Hamburg/Kiel/Lübeck, 20251 Hamburg, Germany

**Keywords:** CRP4, nitric oxide, NO-GC, cGMP, cGKI, PKG, VSMC, vascular tone, blood pressure regulation, Ca^2+^-sensitivity, myofilament

## Abstract

The cysteine-rich LIM-only protein 4 (CRP4), a LIM-domain and zinc finger containing adapter protein, has been implicated as a downstream effector of the second messenger 3′,5′-cyclic guanosine monophosphate (cGMP) pathway in multiple cell types, including vascular smooth muscle cells (VSMCs). VSMCs and nitric oxide (NO)-induced cGMP signaling through cGMP-dependent protein kinase type I (cGKI) play fundamental roles in the physiological regulation of vascular tone and arterial blood pressure (BP). However, it remains unclear whether the vasorelaxant actions attributed to the NO/cGMP axis require CRP4. This study uses mice with a targeted deletion of the CRP4 gene (CRP4 KO) to elucidate whether cGMP-elevating agents, which are well known for their vasorelaxant properties, affect vessel tone, and thus, BP through CRP4. Cinaciguat, a NO- and heme-independent activator of the NO-sensitive (soluble) guanylyl cyclase (NO-GC) and NO-releasing agents, relaxed both CRP4-proficient and -deficient aortic ring segments pre-contracted with prostaglandin F2α. However, the magnitude of relaxation was slightly, but significantly, increased in vessels lacking CRP4. Accordingly, CRP4 KO mice presented with hypotonia at baseline, as well as a greater drop in systolic BP in response to the acute administration of cinaciguat, sodium nitroprusside, and carbachol. Mechanistically, loss of CRP4 in VSMCs reduced the Ca^2+^-sensitivity of the contractile apparatus, possibly involving regulatory proteins, such as myosin phosphatase targeting subunit 1 (MYPT1) and the regulatory light chain of myosin (RLC). In conclusion, the present findings confirm that the adapter protein CRP4 interacts with the NO-GC/cGMP/cGKI pathway in the vasculature. CRP4 seems to be part of a negative feedback loop that eventually fine-tunes the NO-GC/cGMP axis in VSMCs to increase myofilament Ca^2+^ desensitization and thereby the maximal vasorelaxant effects attained by (selected) cGMP-elevating agents.

## 1. Introduction

The vasculature is an integral part of the organism that maintains a proper blood flow, and thus, the supply of the organs with oxygen and nutrients [1,2]. Defects in vascular biology contribute to hypertension leading to cardiovascular diseases (CVD), such as stroke, peripheral artery disease, myocardial infarction, and heart failure [3,4]. The vascular tone is affected by the contractile status of vascular smooth muscle cells (VSMCs), representing the predominant cell type in the media layer of arteries. Excessive vasoconstriction, as well as impaired vasorelaxation, have been linked to the pathophysiology of hypertension [5,6]. In this context, nitric oxide (NO) plays a key role in the regulation of vascular tone. Accordingly, a decrease in NO bioavailability, due to endothelial dysfunction, and thus, abnormal tension of vessels, is common to multiple diseases, including hypertension [7,8,9].

An important signaling molecule downstream of NO is the second messenger 3′,5′-cyclic guanosine monophosphate (cGMP). Cyclic GMP is generated by NO-sensitive (soluble) guanylyl cyclases (NO-GC *aka* GC or sGC) upon NO binding [10]. Alternatively, cGMP is produced by membranous or particulate guanylyl cyclases (pGC) that are mainly represented by GC-A and GC-B and activated by atrial natriuretic peptide (ANP), B-type natriuretic peptide (BNP), and C-type natriuretic peptide (CNP), respectively [11,12,13]. Cyclic GMP, in turn, activates the cGMP-dependent protein kinase type I (cGKI), the most important effector downstream of the NO/NP/cGMP cascade in multiple organs and cell types of the cardiovascular system, including vascular smooth muscle cells (VSMCs) [14]. Both isoforms of the kinase, cGKIα and cGKIβ, produced by the same gene (Prkg1) by two alternative promoters, are present in VSMCs [15,16,17,18]. Activation of cGKIα/β causes phosphorylation of downstream targets, that are, for instance, involved in VSMC relaxation [19]. Cyclic GMP signals are terminated by phosphodiesterases (PDEs), such as PDE5, which is known to degrade the phosphodiester bond in the second messenger cGMP and subsequently dampen NO/NP/cGMP-stimulated cGKI activity in VSMCs [20,21,22]. Besides cGMP degradation, spatiotemporal control of cGMP signals is achieved by the export of cGMP to the extracellular space through, for instance, the ABC transporter multidrug resistance-associated protein 4 (MRP4) [23]. Moreover, feedback inhibition has been discussed as another potential mechanism that fine-tunes NO-evoked cGMP accumulation, which was achieved by cGMP/cGKI through modulating the phosphorylation status of NO-GC [24].

The clinical relevance of the NO/NP/cGMP signaling cascade is widely accepted, as this pathway represents an emerging number of pharmacological targets [25,26,27]. For instance, NO-releasing compounds, such as glyceryl trinitrate, are used to relieve the chest pain caused by angina pectoris attacks [28], inhibitors of PDE5 are in use for the short-term treatment of erectile dysfunction [29], riociguat, a heme-dependent stimulator of NO-GC, is prescribed in patients with pulmonary hypertension [30], and vericiguat, another modulator of NO-GC activity, was recently approved by the FDA and EMA for patients with symptomatic chronic heart failure [31,32].

Investigating genetically modified mice lacking either NO-GC or cGKI revealed that the cGMP cascade is involved in neuronal, metabolic, gastrointestinal, as well as multiple cardiovascular functions, and plays a key role in regulating blood pressure (BP) [33,34,35,36,37,38,39,40,41]. Accordingly, NO-induced vasorelaxation *in vivo* was shown to be mediated by NO-GC and cGKI function, and mice lacking either the cyclase or the kinase developed early-onset hypertension [42,43]. Besides potential effects on cardiac and renal functions, NO-GC/cGMP/cGKI was shown to facilitate the relaxation of VSMCs. Mechanistically, this change in VSMC tone is achieved (i) by reducing the cytoplasmic free calcium ((Ca^2+^)_i_), for instance, by inhibiting phospholipase C (PLC) and inositol 1,4,5-trisphosphate (IP_3_) formation, (ii) by de-/phosphorylation of regulatory proteins involved in the Ca^2+^ desensitization of the myofilaments, and (iii) by membrane hyperpolarization [19]. Cellular targets implicated in mediating these effects of the cGMP pathway in VSMCs are regulatory myosin phosphatase targeting subunit 1 (MYPT1) [44,45], the inositol trisphosphate receptor (IP_3_R) [46,47], as well as the IP_3_R-associated cGMP-kinase substrate (IRAG) [48,49], Ras homolog family member A (RhoA) [50,51], the calcium and voltage-activated K^+^ channel of big conductance (BK_Ca_) [52], regulator of G-protein signaling 2 (RGS2) [53,54], and the vasodilator-stimulated phosphoprotein (VASP) [55,56].

Recently, the cysteine-rich LIM-only protein 4 (CRP4, Gene name: *Crip2*) was described as a novel substrate of cGKI in cardiomyocytes [57], enteric SMC [58], and neurons of the spinal cord [59]. CRP4 was previously termed CRP2 and later renamed to avoid confusion with the smooth muscle development and differentiation cofactor CRP2/smLIM [60,61,62,63]. CRP4 consists of two LIM-domains which are both composed of two zinc fingers. A unique RKTS motif has been identified in the linker region positioned between the LIM-domains of CRP4. This amino acid-sequence, which is not present in the highly homologous proteins CRP1 to 3 [64,65,66], is a putative phosphorylation site for cGKI [58]. Although the cGKI-mediated phosphorylation at the respective serine at position 104 has been confirmed *in vitro* in different cell systems [58,61], the physiological functions of CRP4, as well as the role of this adapter protein for the cGMP pathway in the human or murine organism, is largely unknown. Recently, we observed an amplified cardiac remodeling response in CRP4-deficient mice that received the neurohormone angiotensin II (Ang II). Lack of CRP4 resulted in increased cardiac mass and significant interstitial fibrosis, as well as in a functional decline of the heart during chronic Ang II exposure. These effects were at least partly mediated through the direct interaction of CRP4 with cGKI in the remodeled myocardium [57]. In addition to this cardiac phenotype, baseline BP of mice globally lacking CRP4 (CRP4 KO) was lower. This implies that CRP4 is involved in BP homeostasis, although the observed hypotension is somewhat counterintuitive, because genetically engineered mice lacking putative upstream elements, such as NO-GC or cGKI develop higher, not lower, BP [42,43]. Previous *in vitro* studies reported that CRP4 and cGMP/cGKI act in concert with serum response factor (SRF) and GATA6 to regulate the transcription of VSMC specific genes [61]—thereby strengthening the notion that CRP4 and cGKI interact in intact cells as already supposed by their co-localization in rat intestine [58] and the spinal cord [59]. 

The present study uses mice with a targeted deletion of CRP4, as well as intact vessels and VSMCs derived from this animal model. We applied these models aiming to assess the involvement of the CRP4 and the NO/cGMP/cGKI signaling cascade in the cellular and vascular events controlling BP. 

## 2. Results

### 2.1. Expression Pattern of Vascular CRP4 and Different Components of the NO-GC/cGMP/cGKI Pathway

**Figure 1:**
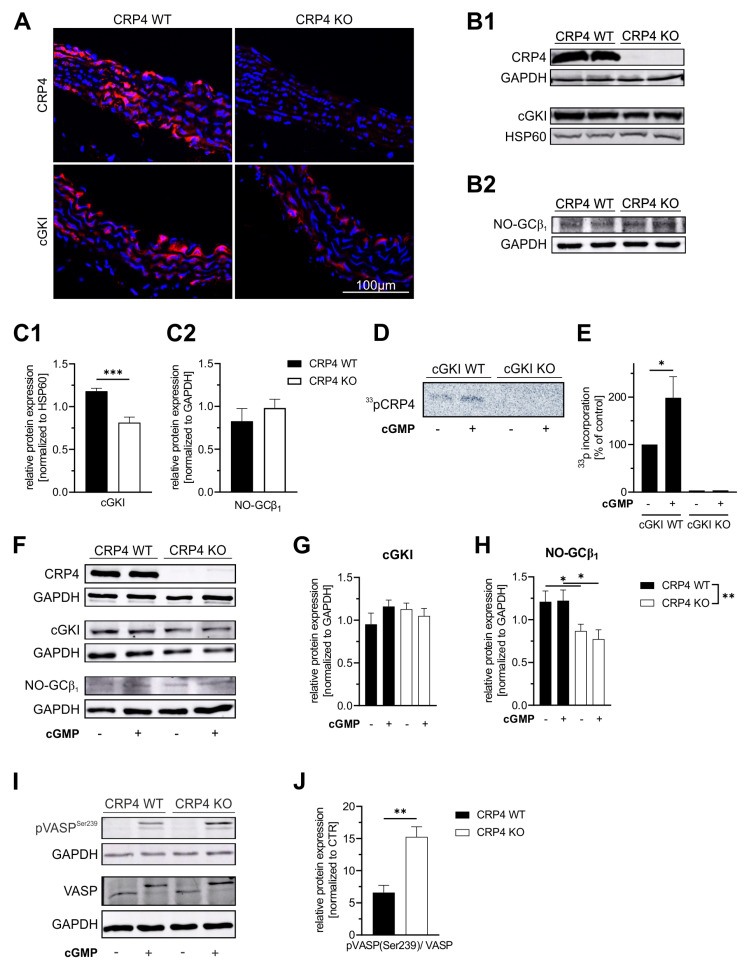
*The LIM-only protein CRP4 status of VSMCs affects the NO-GC/cGMP/cGKI axis*. (**A**) Immunofluorescence (IF) staining of CRP4 WT and global CRP4 KO aortic cryosections demonstrate expression of CRP4 and cGKI in the medial layer of the aorta. As expected, CRP4 was not detectable in CRP4 KO tissue, while the IF signal of cGKI was weaker in the absence of CRP4. (**B**) Western Blot analysis of protein lysates obtained from CRP4 WT and CRP4 KO aorta (**C**) confirmed a significantly lower cGKI protein abundance in CRP4 KO aorta (**B1**,**C1**), whereas the expression of NO-GCβ_1_ was not altered (**B2**,**C2**). Heat shock protein 60 (HSP60) and Glyceraldehyde-3-phosphate dehydrogenase (GAPDH) were used as loading controls. Data represent means ± SEM with n = 8 per genotype (cGKI) and n = 4 per genotype (NO-GCβ_1_), *** *p* < 0.001, *two-*tailed students *t*-test. (**D**,**E**) Autoradiography reveals incorporation of ^33^P from *(*γ-*^33^P*)-ATP into CRP4 only in 8-pCTP-cGMP (100 µM) stimulated aortic tissue expressing cGKI. Data represent means ± SEM with n = 6 per genotype and condition, * *p* < 0.05, two-tailed students *t*-test. (**F**–**H**) In VSMCs (P0), cGKI protein levels did not differ between genotypes at baseline or upon short-term exposure (30 min) to the membrane-permeable cGMP analog 8-Br-cGMP (1 mM). In contrast, the β_1_ subunit, which is essential for NO-GC catalytic activity, was expressed at significantly lower levels in the absence of CRP4 in ±8-Br-cGMP treated lysates obtained from VSMCs. GAPDH was used as a protein loading control. Data represent means ± SEM with n = 6–8 per genotype and condition, ** *p* < 0.01, * *p* < 0.05, two-way ANOVA and Holm-Šídák’s multiple comparisons test used as a post hoc test. (**I**,**J**) Phosphorylation of vasodilator-stimulated phosphoprotein (VASP) at position Ser-239, the cGKI-preferred site, was significantly increased in CRP4 KO in comparison to CRP4 WT VSMCs treated for 30 min with 8-Br-cGMP (1 mM). GAPDH was co-detected to verify equal loading of the gel, and all data were normalized to basal conditions (without 8-Br-cGMP stimulation). Data represent means ± SEM with n = 6 per genotype, * *p* < 0.05, ** *p* < 0.01, two-tailed students *t*-test.

To affirm previously published data regarding potential sources of CRP4 [58,61], we assessed aortic sections derived from CRP4-deficient mice in comparison to respective littermate controls (CRP4 WT) [57]. Immunofluorescence (IF)-based visualization revealed for CRP4 high abundance in the medial layer of the vessel, whereas tissue obtained from CRP4 KO mice remained unstained (Figure 1A). The observed CRP4 expression pattern largely overlapped with IF signals obtained for the cGKI protein, which were enriched in the same layer of the vessel wall [67]. Interestingly, overall levels of cGKI appeared to be lower in the CRP4-deficient aorta (Figure 1A), a finding that was confirmed by Western Blot analyses of protein lysates obtained from whole CRP4 WT and CRP4 KO aorta (Figure 1(B1,C1)). In contrast to cGKI, differences in the amount of the essential β_1_ subunit of NO-GC could not be detected between the two genotypes (Figure 1(B2,C2)). To clarify, whether CRP4 is a substrate of cGKI in “intact” tissue, we performed *in vitro* phosphorylation experiments using *(*γ-*^33^P*)-ATP. Upon permeabilization, aortic segments from cGKI-proficient and -deficient mice were stimulated in the absence or presence of 8-pCPT-cGMP, a membrane-permeable cGMP-analog. Subsequent immunoprecipitation with CRP4-specific antibodies and phospho-imaging (modified from [58]) revealed *^33^P* incorporation, which occurred in a cGMP-dependent manner exclusively in the presence of cGKI. Moreover, the amount of incorporated *^33^P* was significantly increased in response to the 8-pCPT-cGMP treatment, while no CRP4-specific phosphorylation signals were detectable in cGKI KO vessels under these conditions (Figure 1D,E).

Next, we analyzed cultured VSMCs (Passage 0 (P0)) from the aorta of CRP4 WT and CRP4 KO mice with respect to their CRP4, cGKI, and NO-GC protein content by immunoblotting (Figure 1F). In comparison to the aortic lysates (Figure 1B,C), cGKI and NO-GCβ_1_ levels were distinctly affected in isolated VSMCs. While cGKI protein levels did not differ between both genotypes (Figure 1G), the amount of NO-GCβ_1_ was significantly reduced in VSMCs lacking CRP4 (Figure 1H), suggesting that NO-GCβ_1_/cGKI is affected by the CRP4 status and the *in vitro* culture conditions. Stimulation of P0 VSMCs with the membrane-permeable cGMP-analog 8-Br-cGMP did not impact total NO-GCβ_1_ or cGKI levels (Figure 1F–H). Finally, IF analyses with specific antibodies imply that NO-GCβ_1_, cGKI, and CRP4 are spatially colocalized in cultivated VSMCs (Supplementary Figure S1). Remarkably, decreased cGKI (Figure 1B,C) expression levels in the aorta and diminished NO-GCβ_1_ (Figure 1F,H) expression levels in VSMCs, respectively, in the absence of CRP4 resulted in higher levels of vasodilator-stimulated phosphoprotein (VASP) Ser-239 phosphorylation after 8-Br-cGMP stimulation, while total levels of VASP remained unchanged (Figure 1I,J). VASP is considered as an ubiquitously expressed endogenous substrate for cGKI [67,68]. By measuring pSer-239, a residue that is specifically recognized and phosphorylated by cGMP*/*cGKI, VASP provides a valuable biomarker to monitor the activity of the cGMP axis in intact cells. 

In summary, these findings strongly support the notion that CRP4 is a substrate of cGMP/cGKI in VSMCs and in the vasculature. Interaction between CRP4 and the cGMP axis may occur at the level of the putative phosphorylation site at Ser-104 within the RKTS motif or through alternative mechanisms. Lack of CRP4 impacts the NO-GC/cGMP/cGKI axis at different levels, eventually increasing phosphorylation reactions mediated by cGKI. Apparently, this increase in kinase activity is only inefficiently counterbalanced by the lower NO-GC and cGKI expression levels seen in the CRP4 negative VSMCs and vasculature.

### 2.2. cGMP Via CRP4 Affects Norepinephrine-Induced (Ca^2+^)_i_ Signals in VSMCs 

**Figure 2:**
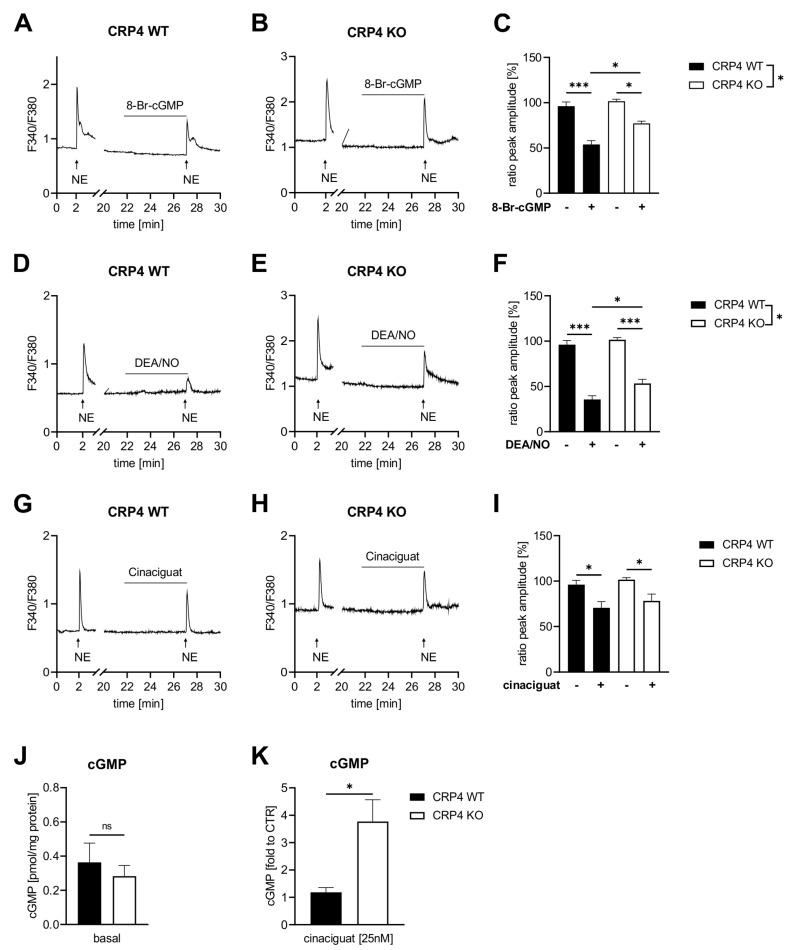
*NO-GC/cGMP oppose agonist-induced (Ca^2+^)_i_ signals through CRP4.* Ratiometric Fura-2 AM Ca^2+^ measurements were performed in norepinephrine (NE) (2 µM) stimulated CRP4 WT and KO VSMCs (P0) in the presence or absence of cGMP-elevating compounds or 8-Br-cGMP. (**A**–**C**) NE-induced (Ca^2+^)_i_ transients were decreased by 8-Br-cGMP, applied 5 min prior to NE, in both genotypes, albeit the decrease in peak height was significantly greater in CRP4 WT VSMCs. The percentage peak height in (**C**) was calculated as the quotient of the NE-induced second peak (+8-Br-cGMP) in relation to the first peak (without 8-Br-cGMP). Data represent means ± SEM with n = 3 (8–9 cells) per genotype, * *p* < 0.05, *** *p* < 0.001, two-way ANOVA, Bonferroni’s multiple comparisons test used as a post hoc test. (**D**,**E**) Pre-incubation (5 min) with the NO-donor diethylamine NONOate (DEA/NO) (10 µM) reduced NE-induced (Ca^2+^)_i_ peak height in CRP4 WT and in KO VSMCs with significant differences between genotypes (**F**). Data represent means ± SEM with n = 5–6 (9–11 cells) per genotype, * *p* < 0.05, *** *p* < 0.001, two-way ANOVA with Bonferroni’s multiple comparisons test used as a post hoc test. (**G**–**I**) Cinaciguat (25 nM) reduced NE-elicited (Ca^2+^)_i_ peaks to a similar extent in both genotypes. Data represent means ± SEM with n = 3 (5–7 cells) per genotype, * *p* < 0.05*,* two-way ANOVA with Holm-Šídák’s multiple comparisons test as a post hoc test. (**J**) Basal cGMP concentrations assessed using a competitive ELISA assay did not reveal differences between CRP4 WT and KO VSMC cultures. Data represent means ± SEM with n = 8 per genotype, two-tailed students *t*-test. (**K**) Cinaciguat (25 nM) induced cGMP formation was significantly elevated in CRP4 KO vs. WT VSMCs. Data represent means ± SEM with n= 4 per genotype, * *p* < 0.05, two-tailed students *t*-test.

Cyclic GMP-induced vasorelaxation is largely due to the suppression of agonist-induced accumulation of cytoplasmic free (Ca^2+^)_i_ in VSMCs [7,42,48]. To test whether cGMP suppresses norepinephrine (NE)-induced (Ca^2+^)_i_ transients in a CRP4-dependent manner, we performed Fura-2 AM based Ca^2+^-measurements [69]. Therefore, cultivated VSMCs (P0) from CRP4 WT and CRP4 KO aorta were stimulated with the α_1_-adrenergic receptor agonist NE. Resulting (Ca^2+^)_i_ transients, which reflect increased transmembrane Ca^2+^-fluxes and release of Ca^2+^ from internal stores, were monitored in real-time for 30 min. The peak amplitude of the (Ca^2+^)_i_ peaks, induced upon stable baseline recording for 2 min, was identical in CRP4 WT and CRP4 KO VSMCs (Figure 2A,B first peak after addition of NE). Once post-NE (Ca^2^^+^)_i_ values had steadied again at the basal level (20 min later), VSMCs were pre-treated with 8-Br-cGMP for 5 min. 8-Br-cGMP significantly decreased the amplitude of subsequently evoked (Ca^2^^+^)_i_ transients by NE in CRP4 WT VSMCs to approx. 54% of the preceding (Ca^2^^+^)_i_ peaks, while in the absence of CRP4 the effect of 8-Br-cGMP on NE-induced (Ca^2^^+^)_i_ peaks was significantly attenuated (Figure 2B–C). These initial (Ca^2+^)_i_ recordings implied that CRP4 was required to decrease NE-dependent (Ca^2+^)_i_ peak formation by exogenously applied 8-Br-cGMP, presumably through cGKI activation [69,70]. To assess whether the (Ca^2+^)_i_ lowering effect of endogenously produced cGMP in agonist-induced (Ca^2+^)_i_ peak amplitudes in VSMCs also requires CRP4, we used the NO-donor DEA/NO, a fast NO-releasing compound that activates NO-GC to produce cGMP. By applying an identical Ca^2+^ imaging protocol DEA/NO-induced a prominent decline in NE-induced (Ca^2+^)_i_ peak amplitudes in both genotypes. Again, however, the resulting (Ca^2+^)_i_ transient reduction by DEA/NO pre-incubation was less pronounced in the absence of CRP4 (Figure 2D–F). This implies that the endogenously stimulated NO-GC/cGMP/cGKI pathway signals via CRP4 to trigger its (Ca^2+^)_i_ lowering properties in NE-stimulated VSMCs. These findings contrast with cinaciguat, an agent that activates NO-GC in a NO- and heme-independent manner, as cinaciguat attenuated the NE-induced (Ca^2+^)_i_ peak amplitudes to a similar extent in CRP4 WT and KO VSMCs (Figure 2G–I). Thus, we conclude that oxidized and/or heme-free NO-GC (NO-GC_ox_) pools in VSMCs, which are largely insensitive to NO, but responsive to cinaciguat, do not efficiently connect with the NE-induced (Ca^2+^)_i_ accumulation via CRP4. Alternatively, the intracellular cGMP formation achieved with cinaciguat differed between both genotypes allowing, for instance, cGKI signaling via alternative Ca^2+^ regulatory proteins in NE-stimulated VSMCs [49,52]. To further investigate this assumption, we directly assessed cGMP at basal conditions and upon cinaciguat exposure in passaged VSMCs (P10–P15). While basal cGMP levels did not differ between genotypes (Figure 2J), cinaciguat-stimulated cGMP formation was increased by approximately 3.8-fold in the absence of CRP4 (Figure 2K). 

Together, 8-Br-cGMP and DEA/NO need functional CRP4 to oppose the agonist-induced Ca^2+^ response in VSMCs. Moreover, lack of CRP4 appears to improve the responsiveness of the cellular NO-GC pool to cinaciguat, an agent that preferentially activates NO-GC_ox_. This cinaciguat-induced cGMP formation, however, does not seem to be involved in the regulatory roles of CRP4 on agonist-induced changes in (Ca^2+^)_i_.

### 2.3. Lack of CRP4 Facilitates Vasorelaxant Actions of the NO-GC/cGMP Cascade

Ca^2+^-measurements revealed, at least partly, an impaired ability of CRP4 KO VSMCs to reduce agonist-induced Ca^2+^-transients via the cGMP pathway (Figure 2). This is counterintuitive with a previous finding, implying that CRP4-deficient mice exhibit a mild, but significant hypotension *in vivo* [57], although BP regulation *in vivo* involves baroreceptors, nervous and the endocrine systems, renal and cardiac functions in addition to changes in vascular contractility, i.e., the agonist-induced and cGMP-modulated Ca^2+^ response of VSMCs. 

**Figure 3:**
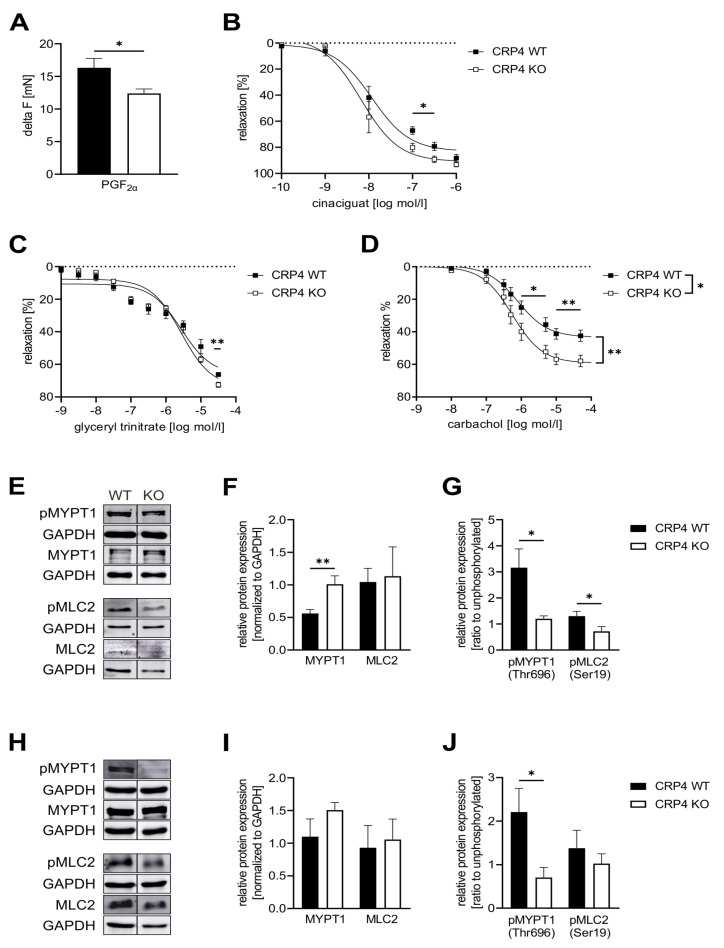
*Lack of CRP4 facilitates Ca^2+^ desensitization mechanisms and cGMP-mediated effects on vascular tone.***(A**) Maximal contraction (delta F (mN)) elicited by Prostaglandin F2α (PGF_2α_) (7.5 µM) was significantly reduced in CRP4 KO vs. CRP4 WT aortic rings equilibrated in an organ bath. Data represent means ± SEM with n = 8–12 per genotype, * *p* < 0.05, two-tailed students *t*-test. (**B**–**D**) Dose-dependent relaxation of aortic ring segments was examined upon precontraction with PGF_2α_ for (**B**) cinaciguat (0.1 nM to 1 µM), (**C**) glyceryl trinitrate (1 nM to 10 mM) and (**D**) carbachol (1 nM to 10 mM)). Continuously, maximal vasorelaxation was significant improved in CRP4 KO aortic ring segments. Relaxation is expressed as percent in comparison to the maximal contraction induced by PGF_2α_. Data points represent means ± SEM with n = 4 per genotype for cinaciguat, n = 5–6 per genotype for glyceryl trinitrate, and n = 6–9 per genotype for carbachol. * *p* < 0.05 or ** *p* < 0.01 represent the statistical difference in responses between the presence, and the absence of cinaciguat, glyceryl trinitrate, or carbachol to PGF_2α_ contracted rings, two-tailed students *t*-test, two-way ANOVA with an uncorrected Fisher’s LSD used as a post hoc test. (**E**–**G**) Western Blot analysis of (**E**) total myosin phosphatase target subunit 1 (MYPT1), pMYPT1 (Thr-696), total myosin light chain 2 (MLC2), and pMLC2 (Ser-19) levels in CRP4 WT and KO VSMCs co-detecting GAPDH in the same protein lysates to demonstrate equal loading of the gels. (**F**) Quantification of the immunoblots revealed that the MYPT1 protein was significantly more abundant in CRP4-deficient protein samples, while total MLC2 did not differ between genotypes. (**G**) Phospho-specific antibodies for Thr-696 in MYPT1, as well as Ser-19 in MLC2, demonstrated significantly less phosphorylation of these regulatory sites of Ca^2+^ sensitivity in protein lysates obtained from CRP4 KO vs. WT VSMCs. Data represent means ± SEM with n = 9–10 per genotype for MYPT1, n = 4 per genotype for pMYPT (Thr-696), n = 6 per genotype for MLC2 and n = 6 per genotype for pMLC2 (Ser-19). * *p* < 0.05 and ** *p* < 0.01 represent the statistical difference between genotypes, two-tailed students *t*-test. (**H**–**J**) Total MYPT1, pMYPT1 (Thr-696), total MLC2, and pMLC2 (Ser19) levels were determined in lysates obtained from the whole aorta using GAPDH as a loading control. (**I**) Consistent with the isolated VSMC lysates Western Blot analyses, a clear, but not significant, increase in MYPT1 protein expression and (**J**) decrease in pMYPT1 at Thr-696 were detected in CRP4 KO aorta tissue, whereas MLC2 and pMLC2 expression were not altered between the genotypes. Data represent means ± SEM with n = 4 per genotype. *p < 0.05 represent the statistical difference between genotypes, two-tailed students *t*-test.

We next performed organ bath experiments with aortic ring segments obtained from CRP4 WT and CRP4 KO mice to address more directly whether vascular CRP4 contributes to relaxation or inhibition of the contractile response. First, maximal contraction of the mounted vessels was elicited by prostaglandin F_2α_ (PGF_2α_). This increase in tone was significantly elevated in CRP4 WT rings. Apparently, CRP4 deficiency impedes contractile force generation, which is reflected by significantly reduced delta values calculated from the difference between the basal and maximally contracted status of the vessel (Figure 3A). Next, aortic ring segments, pre-contracted with PGF_2α_, were relaxed by the addition of different agents acting on the NO-GC/cGMP pathway. Relaxation was related to the respective maximal contraction level recorded in CRP4 WT and KO (Figure 3A). Cinaciguat (Figure 3B and Supplementary Figure S2A), the NO-donor glyceryl trinitrate (GTN) (Figure 3C and Supplementary Figure S2B), as well as carbachol, which raises the endogenous release of NO from the endothelium (Figure 3D and Supplementary Figure S2C), significantly enhanced the maximal extent of relaxation in aortic ring segments lacking CRP4 as compared to CRP4 WT. In this regard, CRP4 seems to play a more prominent role in the vasorelaxant signaling induced by carbachol in PGF_2α_ pre-contracted vessels (Figure 3D), while differences in dose-response curves, as well as the maximal relaxation, were less profound between genotypes when aortic ring segments were treated with GTN or cinaciguat (Figure 3B–C). Thus far, results obtained from aortic tissue preparations *in vitro* indicate that CRP4 diminishes the NO/cGMP/cGKI-induced vasorelaxation, and thus, lack of vascular CRP4 allows the respective cascade to exert its full vasorelaxant reactions.

The finding that CRP4 inhibits in a cGMP-dependent manner also the vasoconstrictive signaling of an increase in (Ca^2+^)_i_ (Figure 2), at least at first sight, appears to conflict with the improved relaxation seen in agonist pre-contracted vessels lacking CRP4 (Figure 3B–D). To resolve this apparent disparity, we examined regulatory proteins that are directly involved in muscle contraction and in Ca^2+^-sensitivity. Phosphorylation of myosin regulatory light chain 2 (MLC2 *aka* regulatory light chain of myosin (RLC)) at Ser-19 is known to increase contractile force in VSMCs without a concomitant increase in intracellular (Ca^2+^)_i_ levels [71,72]. Inhibition of VSMC contraction through cGMP-mediated mechanisms involves phosphorylation of the regulatory targeting subunit 1 (MYPT1) of myosin light chain phosphatase (MLCP) at Ser-695 [44,73]. MYPT1 phosphorylation at this site via cGMP/cGKI disinhibits MLCP activity by preventing phosphorylation of the adjacent inhibitory Thr-696. Subsequently, this allows MLCP to dephosphorylate MLC2 resulting in relaxation [74]. Immunoblotting revealed significantly higher levels of total MYPT1 in CRP4 KO-derived VSMC protein lysates, while the phosphorylation at the inactivating Thr-696 was significantly decreased (Figure 3E–G). This, in turn, indicates an increase in MLC phosphatase activity. In line with this finding, pMLC2 protein levels, but not total MLC2, were significantly reduced in the absence of CRP4 (Figure 3E–G). The same trends were also seen in whole aortic protein lysates, although differences were statistically significant only for the phosphorylation of MYPT1 at Thr-696 (Figure 3H–J). These changes in the phosphorylation status of MLC2 and MYPT1 in isolated VSMCs seem to resemble the situation in the intact aorta, and therefore, explain well why the maximal contractile response of aortic ring segments is reduced (Figure 3A), while concomitant vasorelaxant actions of cGMP-elevating compounds are increased in CRP4 KO aorta. This appears even more interesting because vasorelaxation was attained although agonist-induced Ca^2+^ levels remained higher in CRP4-deficient VSMCs treated with, e.g., 8-Br-cGMP (Figure 2C). Furthermore, we also tested additional regulatory proteins that are directly involved in muscle contraction and in Ca^2+^-sensitivity, e.g., MLCK, rho-associated coiled-coil-forming kinase subtype 2 (ROCK2), and the phosphorylation-dependent inhibitory protein for MLCP protein phosphatase 1 regulatory subunit 14 (CPI-17). MLCK levels did not differ between aortic lysates obtained from CRP4 WT and KO mice (Supplementary Figure S3A,B), while the amount of ROCK2, which promotes the Thr-696 phosphorylation of MYPT1, was slightly, but not significantly, elevated in CRP4 KO aorta tissue (Supplementary Figure S3C,D). Albeit speculative, this indicates the potential counter-regulatory upregulation of ROCK2 expression, due to the reduced Thr-696 phosphorylation of MYPT1. Moreover, CPI-17 phosphorylation levels at Thr38 were negligible under our experimental conditions and unaffected by the CRP4 status (Supplementary Figure S3C,E). 

In conclusion, loss of CRP4, at least partly by acting via the ROCK-MYPT1/MLCP-MLC2 pathway, increases myofilament Ca^2+^ desensitization and facilitates agonist-induced cytoplasmic Ca^2+^ accumulation. In the absence of CRP4, this imbalance of (opposing) events regulating vascular tone promotes vessel relaxation induced by cGMP-elevating compounds. 

### 2.4. CRP4 Interferes with cGMP-Dependent Control of Blood Pressure 

**Figure 4:**
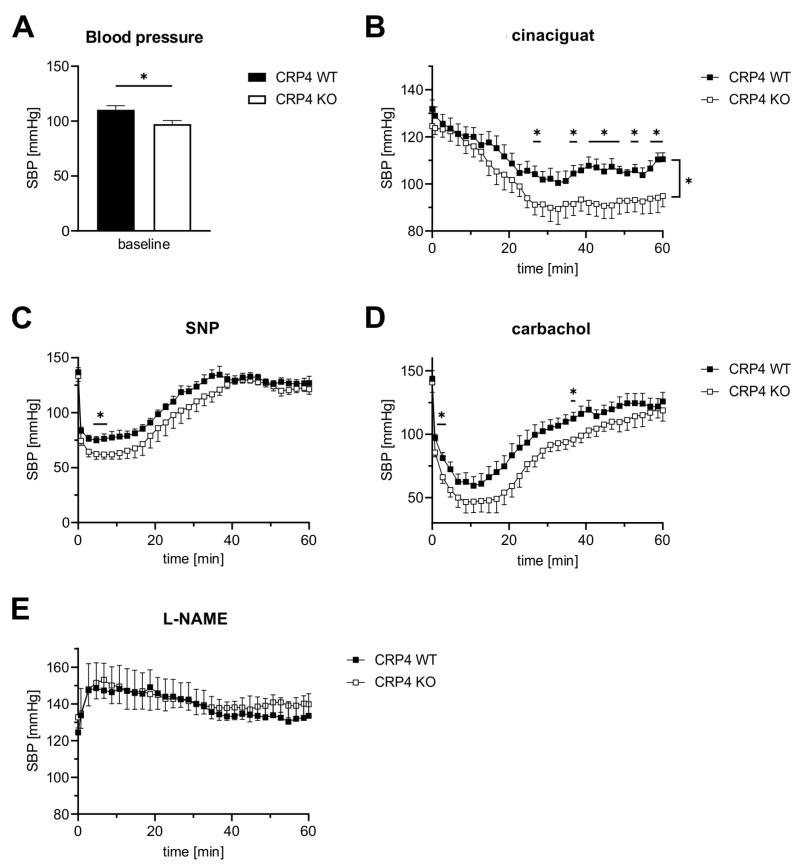
*cGMP-mediated blood pressure regulation involves CRP4.* (**A**) As previously reported [57], basal systolic BP (SBP) of CRP4 KO mice was significantly lower by 13.1 ± 3.15 mmHg than in CRP4 WT mice. * *p* < 0.05 represents the statistical difference between WT and CRP4 KO, two-tailed students *t*-test. (**B**) The NO-GC activator cinaciguat (100 µg/kg bodyweight, i.p.) induced a significantly higher decrease in SBP in CRP4 KO animals. Data points represent means ± SEM with n = 8 per genotype, * *p* < 0.05, repeated-measure ANOVA with an uncorrected Fisher’s LSD used as a post hoc test. (**C**–**D**) Effects of NO-donor sodium nitroprusside (SNP) (2.5 mg/kg bodyweight, i.p.) and the endothelium-dependent vasorelaxant carbachol (0.5 mg/kg bodyweight, i.p.) on SBP were more transient than cinaciguat and resulted in a much faster drop of SBP with consistently significant lower values for CRP4 KO mice. Data points represent means ± SEM with n = 7–8 per genotype for SNP; n = 5 per genotype for carbachol, * *p* < 0.05, repeated-measure ANOVA with an uncorrected Fisher’s LSD was used as a post hoc test. (**E**) Inhibition of endogenous NO-synthase activity by L-NAME (100 mg/kg bodyweight, i.p.) increased SBP to a similar extent in CRP4 WT and KO mice. Data points represent means ± SEM with n = 5–6 per genotype.

Except for some agonist-induced Ca^2+^-transients (Figure 2), our experiments point to pronounced disinhibition of the NO-GC/cGMP axis in the absence of CRP4. If this translates to the *in vivo* situation, BP regulation in CRP4-deficient mice should differ from CRP4 WT. Thus, we performed BP measurements in conscious CRP4 WT and CRP4 KO mice using implantable telemetry devices, which recorded every 30 s for a duration of 15 s [57,75]. First, we confirmed our previous finding of mild hypotonia in CRP4-deficient animals (Figure 4A, Supplementary Figure S4A) [57]. Mean arterial BP 30 min and 60 min after the i.p injection of purified water (aqua ad inject) was not different between genotypes (WT SBP *post_30/60_*: 103.1 ± 2.1 mmHg and 104.9 ± 3.5 mmHg vs. KO SBP *post_30/60_*: 104.8 ± 10.1 mmHg and 102.0 ± 5.1 mmHg) suggesting that short-term BP responses to pre-experimentation handling and injection are intact and not different between genotypes (Supplementary Figure S4B).

Cinaciguat, injected i.p. at a dose of 100 µg/kg bodyweight, significantly reduced SBP in CRP4-deficient mice, while BP responses under these conditions in CRP4 WT were only incremental (Figure 4B). This enhanced BP-lowering effect is congruent with the increased relaxation seen in aortic ring segments lacking CRP4 (Figure 3B) and with the accelerated cGMP formation in cinaciguat-exposed VSMCs (Figure 2K). A much faster and more drastic drop in SBP could be achieved with sodium nitroprusside (SNP, 2.5 mg/kg bodyweight i.p.) (Figure 4C) and carbachol (0.5 mg/kg bodyweight i.p.) (Figure 4D), representing two widely used agents that elicit their effects by increasing vascular NO and the formation of cGMP [76]. The respective changes in SBP affirm CRP4 as an intrinsic regulator of the NO/cGMP axis, because significant lower values could be again observed in CRP4-deficient mice (Figure 4C,D). *Vice versa*, an acute NO-deprivation using L-NAME (100 mg/kg bodyweight i.p.) caused an identical increase in SBP irrespective of the CRP4 genotype of the mice. This suggests that CRP4 functions become only apparent when vascular NO signaling is intact (Figure 4E). Moreover, CRP4 seems to be essential to adequately fine-tune NO-GC/cGMP pathway activation, which may prevent, for instance, overshooting actions of this axis on vascular relaxation causing disorders in BP homeostasis. Together, these unexpected findings identify the cGKI target protein CRP4 as a molecular brake of NO-GC/cGMP affecting vascular tone and thereby BP regulation.

## 3. Discussion

The importance of the NO/cGMP/cGKI signaling cascade in the homeostatic regulation of the vascular system is widely accepted [7]. The ablation of cGMP-generators, such as NO-GC [43] and GC-A [77], provoked increased vascular tonus in gene-targeted mouse models that was also seen *in vivo* and *in vitro* with pharmacological tools that interfere with cGMP signaling [78,79,80]. Cyclic GKI was identified as the major downstream effector of NO/NP-derived cGMP in mediating vascular relaxation [19,42,45,81]. Increases in (Ca^2+^)_i_ and in Ca^2+^ sensitivity of the contractile apparatus determine VSMC contraction [82]. These two interlinked mechanisms are ultimately responsible for the decrease in size of the blood vessel lumen causing high BP. cGMP, in turn, interferes with the vasoconstrictive signaling in VSMCs as this pathway depresses basal and agonist-induced (Ca^2+^)_i_ accumulation and myofilament Ca^2+^ sensitivity [19,83].

**Figure 5:**
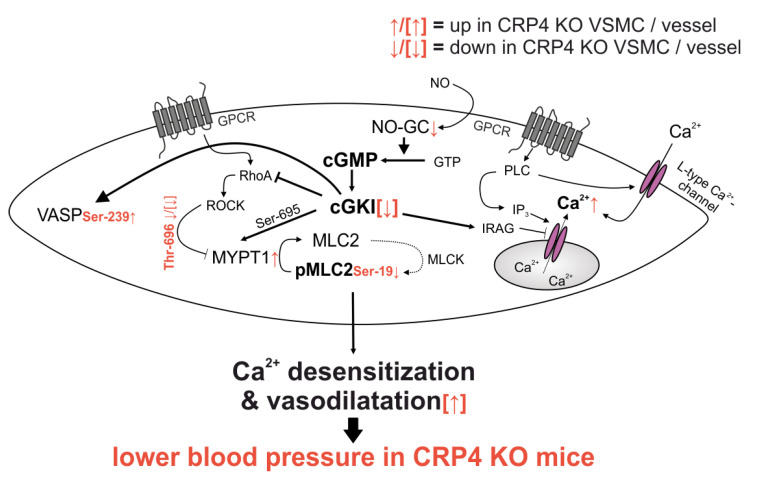
*Synopsis of the altered signaling in CRP4 null VSMCs and vessels.* Lack of CRP4 leads to discrete changes in total protein levels of NO-GCβ_1_, cGKI, and MYPT1 and it causes alteration in the phosphorylation status of pVASP (Ser-239), pMYPT1 (Thr-696) and pMLC (Ser-19), which promotes the Ca^2+^ desensitization of the contractile apparatus independently from the actual (Ca^2+^)_i_ concentration. Combined, these effects are indicative for a disinhibited activity of the NO-GC/cGKI pathway. This results in an improved vascular relaxation and lower BP in mice globally lacking CRP4 (further explanations are given in the main text).

The LIM protein CRP4 was originally identified as a putative cGKI interacting protein in a yeast two hybrid system and in the muscle layer of the rat’s intestine [58]. In general, the LIM-motifs are consensus domains that serve as an interface for protein-protein interactions. Thus, LIM proteins are considered highly versatile adaptor proteins. This feature of LIM proteins is important regarding essential cellular functions, including differentiation, cytosolic organization, gene expression, as well as cell division and motility [64,84,85,86,87]. Because CRP4 is highly expressed in the vasculature [58] and mice globally lacking CRP4 develop hypotension [57], this study aimed to clarify the involvement of CRP4 in VSMC functions affecting vessel contractility, and thus, BP regulation. Also, we addressed a functional connection between the vasodilator response induced by the cGMP axis and CRP4 (Figure 5).

Previous studies on VSMC-like cells suggested that CRP4, through Ser-104 phosphorylation via cGMP/cGKI, affects the expression of smooth muscle (SM)-specific genes, such as SM-α-actin [61]. Incubation of freshly isolated aortic vessels with orthophosphate confirms that CRP4-phosphorylation, presumably at Ser-104, occurs by vascular cGMP/cGKI activation in native tissue (Figure 1D,E). Moreover, by using aorta from cGKI WT and cGKI KO mice [42], we verify that the observed phosphorylation occurred due to 8-pCPT-cGMP-stimulated activation of cGKI and not via the cross-activation of cAMP-dependent protein kinase (PKA), which is known to phosphorylate related consensus sequences in target proteins [58,88]. Although the transcriptional activity of CRP4 was dependent on the ubiquitously expressed SRF and on GATA6 [61], this study suggests that the chronic lack of CRP4 could impact the expression profile of VSMCs. This, in turn, might affect a broad range of proteins involved in, e.g., transmembrane Ca^2+^ fluxes, the release of Ca^2+^ from internal stores, and regulatory proteins of myofilament Ca^2+^ sensitivity as well. Indeed, our approach identified for CRP4 negative VSMCs an increase in MYPT1 abundance (Figure 3E,F). Together with the increase in Thr-696 phosphorylated MYPT1 (Figure 3E,G,H,J) this suggests a diminished Ca^2+^ sensitivity of the contractile apparatus in the absence of CRP4. Moreover, NO-GCβ_1_ and cGKI levels were significantly lower in CRP4-deficient VSMCs and partly also in the aorta, respectively. The differences in cGKI levels, however, disappeared in VSMCs, whereas the diminished NO-GCβ_1_ expression in CRP4 KO mice was only detectable in VSMCs, probably a consequence of the cultivation [89,90,91]. Presently, it is unclear, if these changes in NO-GCβ_1_ and cGKI abundance are due to a CRP4-mediated effect on transcription in smooth muscle. Alternatively, the observed adjustment in NO-GCβ_1_ and cGKI levels could be part of a feedback mechanism that eventually prevents an extensive overactivation of the pathway downstream of cGKI (Figure 1I,J). Indeed, the 8-Br-cGMP-stimulated rise in phospho-VASP (Ser-239) provides evidence for the potential disinhibition of endogenous cGKI activity in the absence of VSMCs CRP4. If this hypothesis is correct, then it would be one function of the endogenous CRP4 protein to fine-tune cGKI activity.

The lower contractile force generation of CRP4 KO aorta segments (Figure 3A), as well as the mild, but significant, hypotonia of CRP4-deficient animals (Figure 4A, [57]), are well in line with the concept of a disinhibited cGKI activity. Changes in contractile force generation and BP are linked to agonist-induced vascular (Ca^2+^)_i_ signaling and the Ca^2+^ sensitivity of VSMC myofilaments. Interestingly, cGMP/cGKI, presumably indirectly via Ser-695 phosphorylation [44], activates MLCP to induce Ca^2+^ desensitization, and thus, vasodilatation [74]. Accordingly, loss of CRP4 resulted in a rise of the MYPT1 protein (Figure 3E,F), the regulatory subunit of MLCP, and a reduction in the inhibitory phosphorylation at Thr-696 of MYPT1 (Figure 1G). This site is believed to be less accessible for modification when the adjacent Ser-695 phosphorylation is present [44]. Thr-696 phosphorylation of MYPT1 is promoted by pro-contractile ROCK [50,92] leading to enhanced MLC2 phosphorylation at Ser-19 [72,93] required for the interaction of myosin with actin filaments, and thus, VSMC contraction [71,94]. *Vice versa*, loss of smooth muscle MYPT1 in mice was associated with, and enhanced MLC2 phosphorylation, an increase in SM contractility, and resulted in high BP [95]. In agreement with this, loss of CRP4 desensitizes (at least partly via cGMP) the myofilaments to Ca^2+^ by changes in MYPT1 levels and MYPT1 phosphorylation, resulting in MLCP activation, while MLCK levels and CPI-17 phosphorylation remained unaltered (Supplementary Figure S2). The effect on MYPT1- and Ca^2+^ sensitivity is consistent with the increase in pVASP/VASP ratio, as well as the vasodilatory and BP-lowering effects seen in the CRP4 negative model (Figure 3B–D, Figure 4B–D), suggesting CRP4 represents an endogenous feedback inhibitor of the cGMP axis.

One important mechanism whereby cGMP leads to relaxation is a decrease in (Ca^2+^)*_i_* in VSMCs [48,52]. Thus, loss of feedback inhibition on NO-GC/cGMP/cGKI, due to CRP4-deficiency, should result in a more drastic reduction of, for instance, NE-induced (Ca^2+^)*_i_* transients. However, 8-Br-cGMP (Figure 2A–C) and NO-releasing drugs, such as DEA/NO (Figure 2D–F), were less effective in opposing agonist-induced (Ca^2+^)*_i_* transients in the absence of CRP4. This increase in (Ca^2+^)*_i_*, appears to conflict with the observed decrease in vascular tone driving low BP in this model. Thus, we can conclude that the decreased Ca^2+^ sensitivity, and not the attenuated suppression of Ca^2+^ transients by cGMP in VSMCs define the vascular contractile response of CRP4-deficient aorta and mice. What mechanisms could contribute to this apparent disparity in myofilament Ca^2+^ sensitivity vs. alterations in (Ca^2+^)_i_? It is, for instance, well accepted that the cGKIβ isoenzyme orchestrates a ternary complex with IRAG and IP_3_R to decrease the release of Ca^2+^ from intracellular stores [48], and it is, thus, tempting to speculate that CRP4 affects the function and/or stability of this complex and thereby NE-induced effects on Ca^2+^ release. This concept fits with the adapter protein functions attributed to LIM-domain proteins and with the agonist-induced formation of a cardiac protein complex comprising cGKI/CRP4 [57]. Thus, NO-GC/cGMP/cGKI may require CRP4 to interact with (Ca^2+^)_i_ opposing mechanisms, while lack of CRP4 promotes more efficiently the (Ca^2+^)*_i_* desensitization of the pathway independently from the actual (Ca^2+^)*_i_* concentration [72,83]. Besides the cGKI-specific recognition site for phosphorylation at Ser-104 [61], CRP4 contains four zinc fingers that can be utilized for multiple protein interactions to assemble relevant signaling components. Through these interactions, a scaffold protein like CRP4 may control dynamic signaling outputs. It, therefore, seems plausible that spatially and temporally confined NO-GC/cGMP/cGKI signaling events are (de)regulated in a different manner in the absence of CRP4.

Although levels of the essential NO-GCβ_1_ subunit are lower in CRP4 KO VSMCs (Figure 1F,H), NO-GCβ_1_ is not altered in CRP4-deficient vessels, while the respective mice showed an increased vasodilatory and BP-lowering response to the heme-independent NO-GC_ox_ activator cinaciguat (Figure 3B and Figure 4B). Conversely, we did not note any genotype-specific differences on the NE-induced (Ca^2+^)_i_ signals that were evoked in the presence of cinaciguat (Figure 2I). We propose several hypotheses that may explain these apparently contradictory findings: (i) We find a significant increase in cGMP formation in cinaciguat treated CRP4 KO as compared to CRP4 WT VSMCs, suggesting that the level of oxidized or heme-free, and thus, cinaciguat-sensitive NO-GC (or NO-GC_ox_), is higher in CRP4-deficient cells or tissue (Figure 2K), even though NO-GCβ_1_ protein abundance is lower. Interestingly, an opposing association between NO-GC expression and function was observed in pulmonary artery smooth muscle cells (PASMCs) of rat models of pulmonary hypertension, due to chronic hypoxia [96,97]. (ii) Cyclic GMP pools generated by NO-GC_ox_ are less efficient in coupling to cGMP/cGKI acting downstream of the axis to oppose transmembrane Ca^2+^-fluxes and/or release of Ca^2+^ from internal stores. This may imply that the cinaciguat-mediated effects on vascular tone and BP are due to changes in MYPT1- and MLC2-mediated Ca^2+^ sensitivity, which decreases in the absence of CRP4. (iii) CRP4 contributes to the oxidative stress level and or the redox regulation of NO-GC *in vivo* rendering a larger fraction of the enzyme responsive to cinaciguat in intact tissue or mice, while in VSMC cultures, cinaciguat-induced cGMP formation does not couple to (Ca^2+^)_i_ handling mechanisms. (iv) Albeit the lower NO-GCβ_1_ protein level in VSMCs, basal cGMP formation was almost identical between CRP4 WT and KO. This suggests that the endogenous CRP4 protein interferes with basal cGMP formation and basal BP (Figure 4A), as well as with NO-GC_ox_ dependent cGMP formation as seen by the cinaciguat-induced changes in vascular tone (Figure 3B) and BP (Figure 4B).

In conclusion, the presented study utilizing a gene knockout model validates the previously identified link between CRP4 and the vascular NO/cGMP/cGKI pathway. The cGKI substrate CRP4 seems to act as a negative feedback regulator on the spatial activity of the cGMP axis. The observed decrease in myofilament Ca^2+^ sensitivity, seen in CRP4-deficient VSMCs, involves relevant changes in MYPT1 and MLC2 phosphorylation, and these discrete changes explain both the reduced maximal force generation of CRP4 KO derived aortic segments and the improved BP responses attained by BP-lowering agents acting through cGMP (Figure 5). While these genotype-dependent alterations indicate the disinhibition of the endogenous NO/cGMP/cGKI pathway, 8-Br-cGMP and the NO-donor DEA/NO need functional CRP4 to oppose NE-induced (Ca^2+^)_i_ signals in cultured (and thus, artificially influenced by serum components) VSMCs. Although speculative, this may also suggest that the adapter protein CRP4, presumably by assembling the relevant signaling components, is important to allow for efficient signaling from the cGMP/cGKI system to (Ca^2+^)_i_ control elements in VSMCs. Overall, our findings imply that CRP4 alters/fine-tunes the vascular response to pharmacological compounds acting through cGMP.

## 4. Material and Methods

### 4.1. Animal Care

The animals were kept under temperature- and humidity-controlled conditions in standardized Makrolon type II or III cages on a 12 h light/dark-cycle. Animals had adlibitum access to food (Altromin, Lage, Germany) and water. For the experiments, we used global CRP4-deficient mice (CRP4 KO) and their wild-type littermate controls (CRP4 WT) on an inbred Sv129 background [57,59]. CRP4 WT and KO mouse strains were bred and maintained at the Institute of Pharmacy at the University of Tübingen as described before [57]. Mice lacking cGKI were generated as previously described at the Institute of Pharmacology and Toxicology, Technical University München [42].

### 4.2. VSMC Culture

VSMC cultures were established from male and female mice euthanized by CO_2_-inhalation at 6–8 weeks. The aorta was dissected and cleaned from adventitial tissue. Next, aortic vessels from ~4 individuals per genotype were pooled and cut into pieces (~1 mm) prior to a first enzymatic digestion step (Papain (0.7 mg/mL) from Sigma Aldrich (Schnelldorf, Germany) for 1 h at 37 °C. Following the second enzymatic digestion (Hyaluronidase Type 1 (1 mg/mL) and Collagenase Type 2 (1 mg/mL), both from Sigma Aldrich (Schnelldorf, Germany), under sterile conditions for 25 min at 37 °C. The isolated cells were then centrifuged (300×*g* for 7 min) and resuspended in DMEM Glutamax cell culture medium containing 5% PenStrep and 10% FCS. VSMC cultures were maintained in sterile incubators at 37 °C and 6% CO_2_ and received fresh medium every third day. After approximately 7 days, when cells grew to 80% confluency, subsequent *in vitro* experiments were performed.

### 4.3. Immunohistochemistry

Immunofluorescence staining was performed to determine the localization of proteins in the aorta and isolated VSMCs. Therefore, the experimental material (aortic cryosections or fixed VSMCs) was permeabilized via 15 min incubation with 0.3% Triton-X 100 in PBS. After rinsing the tissue or cells with PBS, the unspecific binding sides were blocked with 10% NDS in PBS for 1 h at room temperature (RT). Then the following primary antibodies were used for immune-detection overnight at 4 °C: CRP4 (1:2000 dilution [59]), VASP (1:500 dilution, Cell Signaling Technology, Danvers, MA, USA), pVASP (Ser-239) (1:500 dilution, Cell Signaling Technology), NO-GCβ_1_ (1:500 dilution; generously gifted by Prof. A. Friebe, Würzburg [98]), and cGKI (1:500 dilution, Cell Signaling Technology). The primary antibody binding was followed by a fluorophore-coupled secondary antibody binding for 1 h at RT. To detect the primary antibody-antigen complexes, we used AlexaFluor^®^488 goat anti-rabbit antibody (1:800 dilution, Life Technologies, Thermo Fisher Scientifc, Waltham, MA, USA) and subsequently covered the slides with PermaFluor (Thermo Fisher Scientific) containing the cell nucleus marker Hoechst (1:1000 dilution). The detection of the fluorescence staining was performed with the Carl Zeiss Axio Imager.M2 connected with the Apotome 2.0 Slider.

### 4.4. Immunoblot of VSMC Proteins

Western Blot experiments were conducted with isolated VSMCs from CRP4 KO and CRP4 WT mice that were cultured for approximately 7 days to ~80% confluency. For the experiments with 8-Br-cGMP stimulation, VSMCs were serum-starved for 48 h before they were stimulated for 30 min with the membrane-permeable cGMP analog 8-Br-cGMP (1 mM, Biolog, Life Science, Bremen, Germany) or stayed unstimulated. After the stimulation, the VSMCs were immediately lysed with ice-cold SDS lysis buffer (20 mM Tris-HCl (pH 8.3), 0.67% SDS, 23 8 mM β-Mercaptoethanol, phosphatase inhibitor cocktail II and III, cOmplete™ Mini protease inhibitor cocktail) and the protein concentration was measured using the Bradford method. After adjusting the protein concentration to 4 µg/µL, Laemmli buffer was added, and the proteins were stored at −20 °C. Before the protein lysates (60 µg/lane) were separated, and depending on their molecular weight, the protein lysates were denatured at 95 °C for 10 min. For protein separation, we utilized 12.5–17.5% SDS-PAGE and subsequently transferred them to a PVDF membrane (Immobilon; Merck-Millipore, Darmstadt, Germany). Five percent nonfat dry milk in Tris-buffered saline containing 0.05% Tween 20 was used for blocking nonspecific binding sides on the PVDF membrane. The following custom-made generated primary antibodies were utilized for immuno-detection overnight at 4 °C: CPI-17 (1:1000, abcam, Cambridge, UK), pCPI-17 (Thr38) (1:1000, abcam), CRP4 (1:2000 dilution [59]), cGKI (1:1000 dilution; Cell Signaling Technology), NO-GCβ_1_ (1:1000 dilution; generously sponsored by Prof. A. Friebe; Würzburg [98]), VASP (1:1000 dilution; Cell Signaling Technology), pVASP (Ser-239) (1:1000 dilution; Cell Signaling Technology), MYPT1 (1:1000 dilution; Proteintech Europe, Manchester, UK), pMYPT1 (Thr-696) (1:1000 dilution; Cell Signaling Technology), MLC2 (1:500 dilution; Cell Signaling Technology), pMLC2 (Ser-19) (1:1000 dilution; Cell Signaling Technology), MLCK (1:1000, Sigma Aldrich) and ROCK2 (1:1000, Cell Signaling Technology). As loading controls and for protein quantification, we used HSP60 (1:1000 dilution; Santa Cruz Biotechnology, Dallas, Texas, USA) and GAPDH (1:1000 dilution; Cell Signaling Technology). Finally, PVDF membranes were incubated for 1 h at RT with fluorophore-coupled secondary antibodies (Cy5 anti-rabbit in a 1:2500 dilution or Cy3 anti-mouse in a 1:1000 dilution, ECL Plex; GE Life Sciences, Little Chalfont, UK), and antigen-antibody complexes were detected by using the GE Healthcare Amersham Imager.

### 4.5. Organ Bath Experiments to Study Relaxation of Aortic Rings

Animals were euthanized by inhalation of CO_2_ and thoracic aortas were isolated after gently perfusion through heart punction with 1–2 mL Krebs-Henseleit buffer (118.3 mM NaCl; 4.69 mM KCl; 1.87 mM CaCl_2_; 1.0 mM MgSO_4_; 1.03 mM KH_2_PO_4_; 25 mM NaHCO_3_; 11.1 mM D-glucose, pH 7.35) supplied with 5 IE/mL Heparin. Concentration-relaxation curves of aortic tissue were measured as described previously [99]. In brief, the thoracic part of aortas isolated from CRP4 WT and CRP4 KO mice were liberated from fatty tissue, rinsed, and cut into 4 mm segments. The aortic rings were mounted between stainless triangles in an organ bath chamber connected to force transducers to measure isometric tension through a computerized acquisition system (Kent scientific corporation, Torrington, CT, USA; Powerlab, ADInstruments, Spechbach, Germany) and a computer running Labchart 4.0 software (AD Instruments, UK). The organ chambers with a capacity of 20 mL were gassed with 95% O_2_/5% CO_2_ and filled with Krebs-Henseleit buffer. The aortic rings were stretched progressively to achieve a resting tension of 1.0 g. To analyze agonist-induced relaxation responses, the vessels were pre-constricted with 7.5 µM Prostaglandin F2α (PGF_2α_; Sigma Aldrich) to assess their maximal tension. Relaxation responses were evoked by concentration-response curves to cumulatively increasing concentrations of cinaciguat (0.1 nM–250 nM), glyceryl trinitrate (1 nM–30 µM), and carbachol (10 nM–50 µM). The respective tension to each concentration was recorded, and the relaxation was finally calculated with respect to the maximal contraction. 

### 4.6. In Vivo Phosphorylation of CRP4 in Murine Aorta

Aorta from cGKI WT and cGKI-deficient mice [42] were isolated and bisected. Each segment was labelled with (^33^P)-H_3_PO_4_ (200 µCi/mL; ICN) in buffer (in mM: 115 NaCl, 4.7 KCl, 1.18 MgSO_4_, 1.2 KH_2_PO_4_, 3.2 CaCl_2_; NaHCO_3_, 10 glucose; pH 7.4; aerated with 5% CO_2_ in O_2_) for 4 h at 37 °C. One aortic segment was treated with 100 µM 8-pCPT-cGMP (Biolog Life Science) in the presence of the phosphatase inhibitor okadaic acid (100 nM; RBI) 15 min before stopping the labeling period, while the other segment was run as control. Aortic segments were snap-frozen, thawed, and incubated with 200 µL SDS lysis buffer (50 mM Tris, 0.5% SDS; pH 8.0). The lysates were treated at 95 °C for 5 min, incubated with 150 units of DNase I (Roche Diagnostics, Basel, Switzerland) for 15 min at RT, and subsequently diluted 4-fold with RIPA correction buffer (12.5 mM Na_2_HPO_4_, 1.25% Nonidet P-40, 1.25% sodium desoxycholat, 2 mM EDTA, 50 mM NaF; pH 7.2) containing 1 µg/mL aprotinin, 2 mM benzamidine, 100 µM phenylmethylsulfonyl fluoride, 100 nM okadaic acid. After ultra-centrifugation with 100.000× *g* for 30 min at 4 °C, the supernatant was incubated with CRP4 antiserum for 2 h at 4 °C, followed by protein A-Sepharose (Sigma Aldrich; 4 °C, 2 h) which was pre-absorbed and pre-equilibrated with 0.1 mg/mL bovine serum albumin in RIPA buffer (10 mM Na_2_HPO_4_, 1% Nonidet P-40, 1% sodium desoxycholat, 0.1% SDS, 150 mM NaCl, 2 mM EDTA, 50 mM NaF; pH 7.2; containing 1 µg/mL aprotinin, 2 mM benzamidine, 100 µM phenylmethylsulfonyl fluoride, 100 nM okadaic acid). After incubation, protein A-Sepharose beads were pelleted, and absorbed proteins were eluted with Laemmli buffer prior gel-electrophoresis and blotting onto PVDF membranes. Phosphorylated CRP4 was identified by autoradiography and phospho-image analysis (BAS-1500, Fuji, Raytest). The immunoprecipitated amount of CRP4 was visualized by incubating the PVDF membrane with CRP4 antiserum and tagging it with an alkaline phosphatase-conjugated donkey anti-rabbit IgG (Dianova, Hamburg, Germany; 1:5000 in 1xTBST, 5% BSA, 0.05% sodium azide) (data not shown). For quantification of phospho-luminescence, AIDA 2.0 image analysis program was used. The obtained signals were correlated with the immunoprecipitated amount of CRP4.

### 4.7. Telemetric Blood Pressure Measurement

The measurement of blood pressure, heart rate, and locomotive activity was performed via the Data Science International telemetry acquisition system (DSI, St. Paul, MN, USA) with TA11PA-C10 transmitters in CRP4 WT and CRP4 KO mice. Implantation of the transmitter was previously described [57,100]. In brief, after the animals received an analgesic treatment with ketamine (80 mg/kg bodyweight) and xylazine (10 mg/kg bodyweight), inhalation anesthesia, containing a mixture of oxygen and the anesthetic gas isoflurane (0.5–2%), was applied. The mouse was set on a heat plate (37 °C), and a small ventral, submandibular incision was made to access the left carotid artery and dissect it clear from the surrounding tissue and the nervus vagus. Caudal to the bifurcation, the first permanent ligature was placed and a second reversible ligature proximal of the first one. Thereafter, the carotid artery was carefully scribed with a cannula, and the catheter was inserted and moved forward to the aortic arch. The catheter was then fixed with two more permanent ligations, and the transmitter was position subcutaneously in the right flank area between the front and rear legs. Animals could recover for a minimum of seven days. Then, blood pressure was measured on three consecutive days, and the mean of these three measurements was used for statistical analyses. Before injection, the basal blood pressure was measured for 30 min (15 s during a 30 s-time interval). Then, aqua ad injectabilia (200 µL) was injected (i.p.), and the blood pressure was measured for a further 60 min (15 s during a 30 s-time interval). In the following weeks cinaciguat (100 µg/kg bodyweight, modified from [101]), sodium nitroprusside (2.5 mg/kg bodyweight [102]), carbachol (0.5 mg/kg bodyweight [103]) and L-NAME (100 mg/kg bodyweight [78]) were injected and measured the same way (one substance per week).

### 4.8. Fluorescent-Based Ca^2+^ Measurements in VSMCs

To measure intracellular Ca^2+^ concentrations in VSMCs, we used a fluorescent-based method with the esterified fluorescence marker Fura-2 AM. After the VSMCs grew to 80% confluency in glass dishes, they were serum-deprived for 48 h. Then the cells were incubated with Fura-2 AM (Merck Millipore) for 45 min at 37 °C in the dark. Before the measurement was started, the cells were rinsed for 8 min with Tyrode (NaCl, HEPES, D-Glucose, 0.5 M KCl, 0.12 M MgSO_4_, 1 M CaCl_2_) to remove excessive Fura-2 AM. The measurement at the fluorescence microscope (Axiovert S100, Zeiss, Jena, Germany) started with a 2 min measurement with Tyrode followed by the first norepinephrine (NE, 2 µM) (Sigma Aldrich) stimulation for 5 s. Accordingly, the cells were rinsed again with Tyrode for 20 min and then they were incubated for 5 min with either 8-Br-cGMP (1 mM) (Biolog Life Science), cinaciguat (25 µM), or DEA/NO (10 µM) (Sigma Aldrich) prior to a second NE stimulation. The calculation of the measured fluorescence signal was performed with ImageJ.

### 4.9. Cyclic GMP ELISA Measurements

The calculation of cGMP concentration in VSMCs was conducted with a competitive ELISA kit (Cyclic GMP ELISA kit, Cayman Chemical, Ann Arbor, Michigan, USA). VSMCs were isolated and cultured in 10 cm dishes. Before the cells were lysed with 500 µL ice-cold 100% ethanol, they were stimulated with cinaciguat (25 nM) (Sigma Aldrich) or culture media for the control group for 10 min. Centrifugation for 7 min at 13.000 rpm resulted in a supernatant containing the cyclic nucleotides, which were concentrated by vaporization of the ethanol in a vacuum centrifuge at RT. Determination of the cGMP level were performed following the manufacturer’s description, and the assay was spectrophotometrically evaluated at 405 nm (Sunrise, Tecan, Männedorf, Switzerland). The protein concentration of the remaining protein pellet after the first centrifugation step was measured and used for normalization of the cGMP content.

### 4.10. Statistical Analyses

All data are expressed as means ± SEM. To confirm Gaussian distribution, we performed the Shapiro-Wilk test and Kolmogorov-Smirnov test. All data were normally distributed, and in the case of experimental data consisting of 2 groups the unpaired Student *t*-test was used for statistical analysis. Two-way ANOVA was used for statistical analysis if more than two groups were compared. Holm-Šídák’s multiple comparisons test (Figure 1H and Figure 2I) or Bonferronis’s multiple comparisons test (Figure 2C,E) were used as a post hoc test. In Figure 3B–D, the significance of the curves was calculated by a two-way ANOVA test, and the uncorrected Fisher’s LSD was used as a post hoc test. Repeated-measure ANOVA was applied for Figure 4B–D, and significant single timepoints were calculated with uncorrected Fisher’s LSD as a post hoc test. *p*-values of <0.05 were considered significant, and all statistical analysis was performed with Graph pad prism (Version 9).

## 5. Conclusions and Limitations of the Study

The presented results derive from a constitute KO mouse model, which lacks CRP4 during development in all somatic and germline cells. This global ablation of CRP4 may induce genetic compensatory mechanisms that we could not completely rule out. Indeed, we did find discrete changes in NO-GC and cGKI abundance in CRP4 negative VSMCs and at least partly also in the aorta, respectively, while other components of the pathway were not assessed in detail. In addition, lack of CRP4 might alter the expression of other genes relevant for Ca^2+^ handling and may also induce changes in, for instance, highly related LIM proteins [57,104]. Similar compensatory mechanisms involving members of the CRP family have been identified in cardiac muscle [57] and the vasculature [65]. While primary VSMCs are a widely used surrogate for studying the vascular signaling events regulating tone, maintenance of these cells in culture triggers their switch from a contractile to a proliferative state. This transition, as well as the lack of endothelial cells, may alter the response of the cells to vasoactive agents and is a major weakness of the model.

Although our findings imply that vascular CRP4 must be considered as an endogenous “brake” that prevents “overspill” in NO/cGMP/cGKI signaling, BP is regulated by other mechanisms besides vascular tone, which could contribute to hypotonia in CRP4-deficient mice. Another shortcoming of our study is the lack of a pSer-104 specific CRP4 antibody, which would allow us to directly correlate the activation of the cGMP axis, an increase in Ca^2+^ desensitization of the myofilaments and/or vascular tone with the pSer-104 status of CRP4. Accordingly, neither activating nor inhibiting agents are available that enable us to target CRP4 directly. This is important because acutely provoked alterations in subcellular localization, abundance, or protein-protein interactions could lead to results differing from those of our study, which relies on a chronically depleted CRP4 mouse model.

## Data Availability

The raw data and the analytic methods will be made available to other researchers for purposes of reproducing the results in their own laboratories on reasonable request. To access protocols or datasets contact peter.ruth@uni-tuebingen.de or robert.lukowski@uni-tuebingen.de.

## Supplementary Materials

The following are available online at https://www.mdpi.com/article/10.3390/ijms22189925/s1.
