# Supplementary material for: Cyclic GMP-Dependent Regulation of Vascular Tone and Blood Pressure Involves Cysteine-Rich LIM-Only Protein 4 (CRP4)
Source: Int J Mol Sci. 2021 Sep 14;22(18):9925. doi: 10.3390/ijms22189925 (PMC8466836; doi:10.3390/ijms22189925)
Supplement: Supplementary file 1 [file ijms-22-09925-s001.zip › ijms-1221004-supplementary.pdf]

# Cyclic GMP-Dependent Regulation of Vascular Tone and Blood Pressure Involves Cysteine-Rich LIM-Only Protein 4 (CRP4)

Natalie Längst[1,#], Julia Adler[1,#], Olga Schweigert[2,3], Felicia Kleusberg[1], Melanie Cruz Santos[1], Amelie Knauer[1], Matthias Sausbier[1], Tanja Zeller[2,3], Peter Ruth[1,§], Robert Lukowski[1,§]

[#]*These authors contributed equally to this work*

[1]Department of Pharmacology, Toxicology and Clinical Pharmacy, Institute of Pharmacy, University of Tuebingen, 72076 Tuebingen, Germany
[2]Cardiovascular Systems Medicine and Molecular Translation, University Center of Cardiovascular Science, University Heart & Vascular Center Hamburg, University Medical Center Hamburg-Eppendorf, Clinic of Cardiology, University Heart and Vascular Center, 20251 Hamburg, Germany
[3]DZHK, German Center for Cardiovascular Research, partner site Hamburg/Kiel/Lübeck, 20251 Hamburg, Germany

[§]Corresponding authors:

Robert Lukowski
Experimental Pharmacology
Department of Pharmacology, Toxicology and Clinical Pharmacy
Institute of Pharmacy
University of Tübingen
Tel. +49 7071 29 74550
Fax +49 7071 29 2476
E-mail: robert.lukowski@uni-tuebingen.de

or

Peter Ruth
Department of Pharmacology, Toxicology and Clinical Pharmacy
Institute of Pharmacy
University of Tübingen
Tel. +49 7071 29 76781
Fax +49 7071 29 2476
E-mail: peter.ruth@uni-tuebingen.de

**Supplemental Figures and Legends:** 4

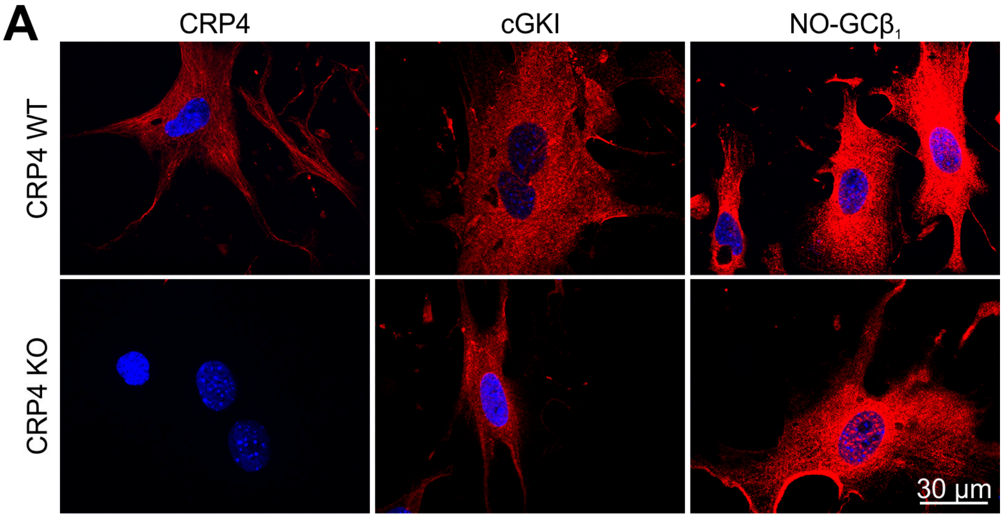


**Figure S1.** *Immunofluorescence (IF) analysis of primary VSMCs.* CRP4, cGKI and NO-GC$\beta_1$ were consistently detectable by antigen-specific primary antibodies and secondary antibodies coupled to fluorescent dyes in CRP4 WT VSMCs. As expected, CRP4 KO VSMCs remained negative for CRP4, while the cGKI and NO-GC$\beta_1$ expression patterns did not differ substantially between genotypes.

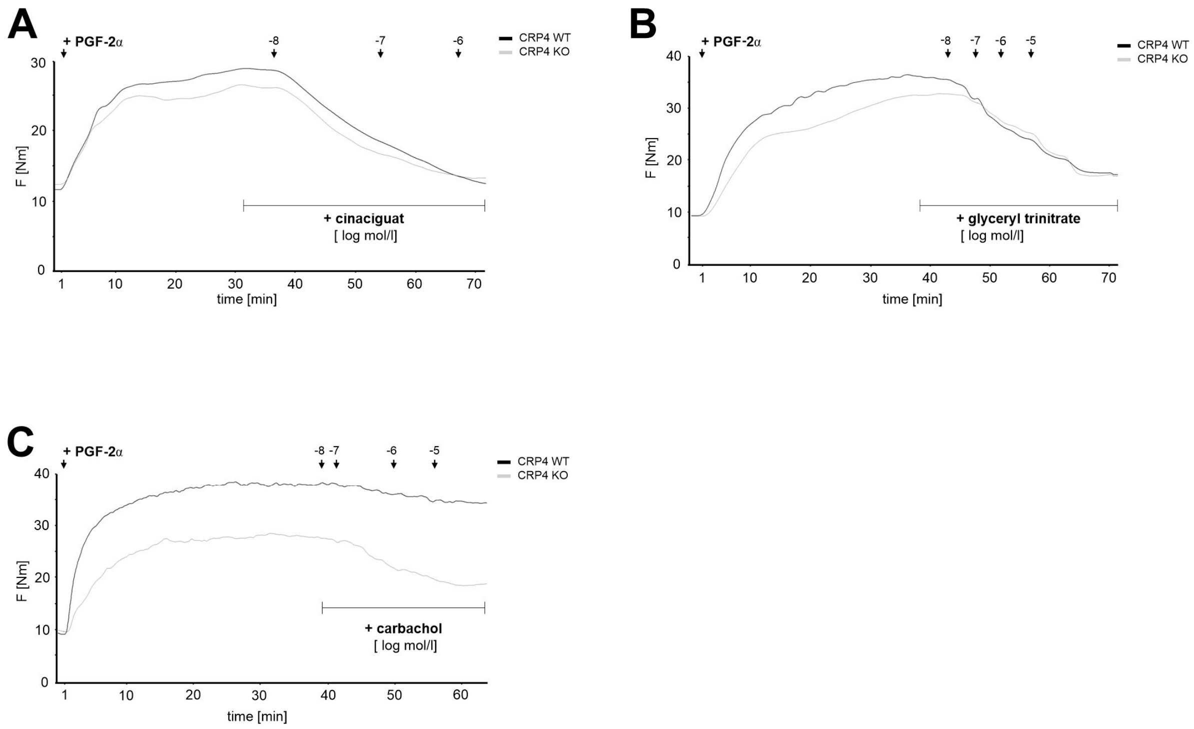


**Figure S2.** *Representative raw records of aortic ring segments exposed to different vasoactive agents.* Representative basic recordings of vascular tone during vasoconstriction and vasodilation of aortic rings. Dose-dependent relaxation of aortic ring segments was examined upon precontraction with PGF2α for cinaciguat [0.1 nM to 1 μM], glyceryl trinitrate [1 nM to 10 mM] and carbachol [1 nM to 10 mM].

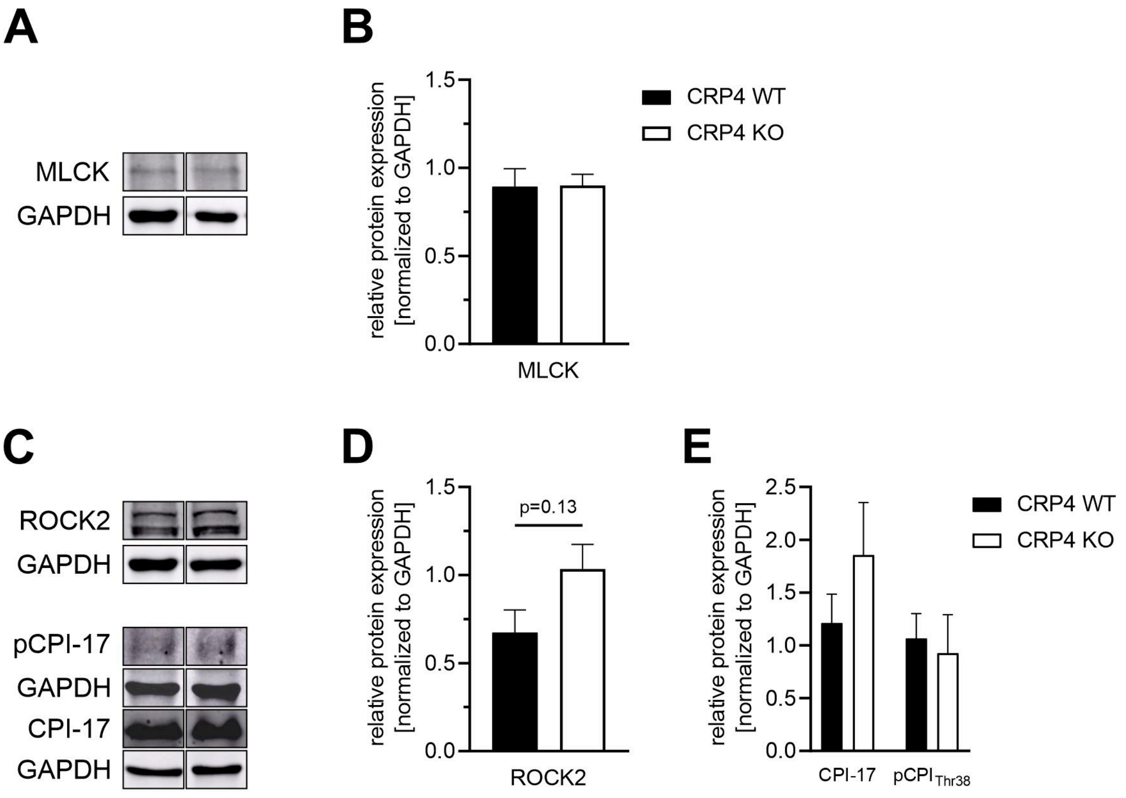


**Figure S3.** *Lack of vascular CRP4 did not alter MLCK, ROCK2 and pCPI-17/ pCPI-17.* (**A-B**) Western Blot analysis of total myosin light chain kinase (MLCK) in CRP4 WT and KO aorta. (**C-D**) The expression of rho-associated coiled-coil-forming kinase subtype 2 (ROCK2) was slightly, but not significantly, increased in CRP4 deficient aorta, (E) while protein kinase C-potentiated protein phosphatase-1 inhibitor (CPI-17) and the activating phosphorylation of CPI-17 at Thr-38 were unaffected by the CRP4 status of the vessel. To demonstrate equal loading of the gels, GAPDH was co-detected in all samples. Data represent means ±SEM with n=4 per genotype.

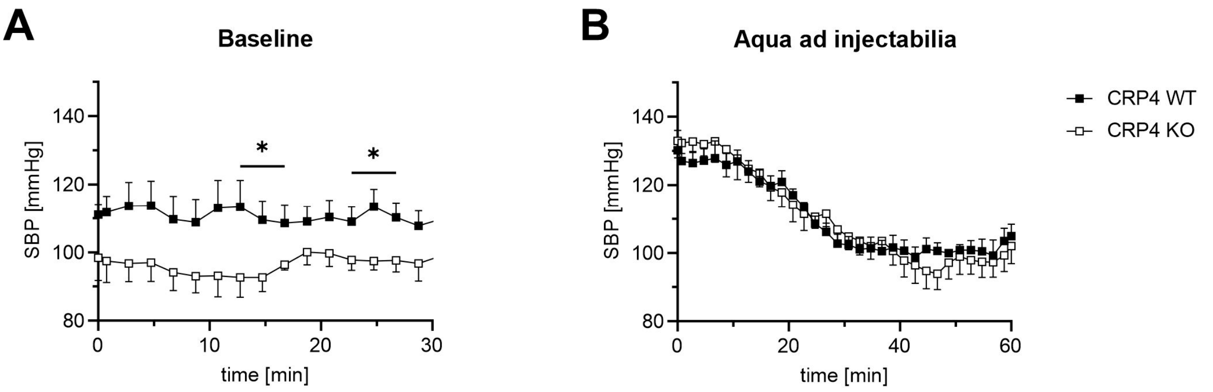


**Figure S4.** *Lack of CRP4 does not alter the "stress-induced" BP response.* (**A**) Basal vascular tone of CRP4 WT and CRP4 deficient animals analysed by telemetric blood pressure measurements demonstrated a mild hypotonia in CRP4 KO animals. Data represent means ±SEM with n=8 per genotype. *P<0.05 represent statistical difference between genotypes, two-tailed students t-test. (**B**) Injection of saline (*aqua ad injectabilia* applied i.p.) reduced the SBP in CRP4 WT and KO animals to a similar extent suggesting that short-term blood pressure responses to pre-experimentation handling and injection, which result in a temporarily "stress-induced" BP increase, are intact in CRP4-deficient animals. Data points represent means ±SEM with n=4-5 per genotype.
